# Microarray Analysis For Expression Profiles of lncRNAs and circRNAs in Rat Liver after Brain-Dead Donor Liver Transplantation

**DOI:** 10.1155/2019/5604843

**Published:** 2019-11-07

**Authors:** Sanyang Chen, Hongbo Fang, Jie Li, Jihua Shi, Jiakai Zhang, Peihao Wen, Zhihui Wang, Han Yang, Shengli Cao, Huapeng Zhang, Hongwei Tang, Wenzhi Guo, Shuijun Zhang

**Affiliations:** ^1^Department of Hepatobiliary and Pancreatic Surgery, The First Affiliated Hospital of Zhengzhou University, No. 1, East Jian She Road, Zhengzhou, Henan Province 450052, China; ^2^Henan Key Laboratory of Digestive Organ Transplantation, Zhengzhou, Henan Province 450052, China; ^3^Open and Key Laboratory of Hepatobiliary & Pancreatic Surgery and Digestive Organ Transplantation at Henan Universities, Zhengzhou, Henan 450052, China; ^4^Zheng Zhou Key Laboratory of Hepatobiliary & Pancreatic Diseases and Organ Transplantation, China

## Abstract

The mechanisms underlying severe liver injury after brain-dead (BD) donor liver transplantation (BDDLT) remain unclear. In this study, we aimed to explore the roles of lncRNAs and circRNAs in liver injury after BDDLT. Rat liver injury was detected in the sham, BD, control, and BDDLT groups. We examined the expression profiles of lncRNAs and circRNAs in the livers of the BDDLT and control group using microarray analysis. The main functions of the differentially expressed genes were analyzed by gene ontology (GO) and KEGG pathway enrichment analysis. In addition, we used bioinformatic analyses to construct related expression networks. Liver injury was aggravated in the BD and BDDLT groups. We found various mRNAs, lncRNAs, and circRNAs that were differentially expressed in the BDDLT group compared with those in the control group. Coding-noncoding gene co-expression (CNC) network analysis showed that expression of the lncRNA LOC102553657 was associated with that of the apoptosis-related genes including HMOX1 and ATF3. Furthermore, competing endogenous RNAs (ceRNAs) network analysis revealed that the lncRNA LOC103692832 and rno_circRNA_007609 were ceRNAs of rno-miR-135a-5p targeting Atf3, Per2, and Mras. These results suggest that lncRNAs and circRNAs play important roles in the pathogenesis and development of liver injury during BDDLT.

## 1. Introduction

In current practice, most liver grafts are obtained from brain-dead (BD) donors [1]. Brain death refers to the irreversible and permanent loss of all function throughout the entire brain, including the brainstem [2]. Previous clinical and experimental studies have shown that a series of complex pathophysiological changes occur during brain death, including hemodynamic instability, increased leukocyte infiltration, hormonal imbalance, induction of cytokines and chemokines, and increased expression of heat shock proteins. These changes reduce the tolerance of liver grafts to the further cold preservation/reperfusion injury and aggravate liver injury after liver transplantation (LT), thus reducing the graft survival rate [3–6]. Despite an increasing number of studies on brain death, to date, there have been few in-depth studies on its mechanism, especially at the genetic level. Furthermore, no precise biomarker has been identified which can predict liver injury following BD donor LT (BDDLT).

Noncoding RNAs are RNA molecules that normally do not encode proteins, but instead functionally regulate protein expression [7, 8]. Noncoding RNAs mainly include long noncoding RNAs (lncRNAs), circular RNAs (circRNAs), and microRNAs (miRNAs) [9]. lncRNAs are transcripts longer than 200 nucleotides which, because of their stability, are ideal biomarkers for disease research. New evidence suggests that lncRNAs play a key role in regulating many cellular biological processes, including cell proliferation, differentiation, apoptosis, migration and invasion, and the cell cycle [10–12]. In recent years, their potential roles in liver injury have been recognized. The lncRNA Gm2199 can promote hepatocyte regeneration and provides protection against liver injury, while the lncRNAs HOTAIR, HULC, and uc003wbd are highly expressed in patients with liver cancer or hepatitis B [13–16]. Recent studies have shown that circRNAs play a key role in the development of human diseases. CircRNAs are a type of RNA formed from a single-stranded, covalently closed loop, which serve mainly as a “sponge” for miRNAs [17, 18]. Additionally, circRNAs can compete with pre-mRNA splicing or affect gene transcription by their association with phosphorylated Pol II [19, 20]. Recently, an increasing number of studies have focused on the role of circRNAs in liver injury [21, 22].

Although Catania et al. investigated changes in mRNA expression during brain death [23]. However, to date, no study has explored the mechanisms by which noncoding RNAs participate in liver injury after BDDLT. Therefore, to investigate the role of noncoding RNAs in liver injury after BDDLT, we measured the differential expression of lncRNAs, circRNAs, and mRNAs in liver tissues after BDDLT, using microarray analysis. Significant differences in expression levels of lncRNAs and mRNAs were further verified by quantitative real-time polymerase chain reaction (qRT-RCR) analysis. In addition, we analyzed related pathways and gene ontology (GO), and furthermore performed network analyses of coding-noncoding gene CNC (CNC), competing endogenous RNAs (ceRNAs), lncRNAs' adjacent coding genes, and TF-lncRNAs enriched with mRNAs using bioinformatic methods. Our study aimed to elucidate the mechanisms of liver injury after BDDLT to help identify biomarkers of liver injury and new therapeutic targets.

## 2. Materials and Methods

### 2.1. Animals and Establishment of BD, Orthotopic LT Model

Male Lewis rats weighing 250–280 g were purchased from Beijing Vital River Laboratory Animal Technology Co. (Beijing, China). All of the experimental procedures were approved by the Animal Ethics Committee of Zhengzhou University.

Brain death was induced by increasing intracranial pressure in a modified, slow, and intermittent fashion [24]. A 3F Fogarty catheter was connected to a pressure pump and then inserted into the epidural space through a hole in the skull. An additional catheter was inserted into the saphenous artery to record the arterial blood pressure, and a blood pressure above 80 mmHg was considered normal. The liver was procured 6 h after brain death induction according to the clinical practice [25, 26].

Livers from BD and nonBD donors were perfused with the University of Wisconsin (UW) preservation solution. The rat models of LT were established using the two-cuff technique without liver artery reconstruction [27]. The anhepatic phase lasted 17–20 min.

Experimental animals were randomized into four groups: sham-operated rats underwent only drilling of the skull and laparotomy, without any other procedure performed; in the BD group, brain death was maintained for 6 h after induction; in the control group, the sham-operated rats were used as donors for orthotopic LT; in the BDDLT group, grafts for orthotopic LT were obtained 6 h after brain death. Serum and tissue samples were obtained before liver harvesting and 12 h after reperfusion.

### 2.2. Biochemistry and Enzyme-Linked Immunosorbent Assay (ELISA)

Serum alanine aminotransferase (ALT) and aspartate aminotransferase (AST) were measured using a fully automated multi-functional serum biochemical analyzer (7600-020, Hitachi, Japan). Malondialdehyde (MDA) and superoxide dismutase (SOD) activity in the liver tissue were detected using an ELISA kit (Jiancheng, Nanjing, China). Liver tissue myeloperoxidase (MPO), liver tissue 3-nitrotyrosine, bile lactate dehydrogenase (LDH), and gamma-glutamyl transferase (GGT) were detected using a different ELISA kit (CUSABIO, Wuhan, China).

### 2.3. TUNEL Fluorescence Assay

From the TUNEL kit, appropriate volumes of reagent 1 (TdT) and reagent 2 (dUTP) were mixed at a ratio of 1 : 9 to cover the tissue. The mixture was added to the tissue with DAPI and then incubated at room temperature in the dark. The results were observed under a fluorescence microscope [24].

### 2.4. RNA Extraction and Quantitative Real-Time PCR Validation

RNA was extracted from tissue using TRIZOL (Invitrogen, America). Synthesis of cDNA was performed with a reverse transcription kit (TaKaRa, Japan) using the Gene Amp PCR System 9700 (Applied Biosystems, America), real-time PCR was performed using SYBR Green PCR Master Mix (TaKaRa) on the Gene Amp PCR System 9700, and the primers used were as described below. The primers of the randomly selected lncRNAs, mRNAs, and internal parameters of GAPDH are shown in Table 1.

### 2.5. Microarray Assay

Microarray analysis was performed by KangChen Bio-tech, Shanghai, PR China. Briefly, Three pairs of liver tissues were harvested 12 h after LT in the BDDLT group and the control group. Differential expression levels of lncRNAs, circRNAs, and mRNAs were measured using microarray analysis techniques [28]. Arraystar Rat LncRNA Microarray V2.0 was used. Sample labeling and array hybridization were performed according to the Agilent One-Color Microarray-Based Gene Expression Analysis protocol (Agilent Technology) with minor modifications. Briefly, mRNA was purified from total RNA after removal of rRNA (mRNA-ONLY™ Eukaryotic mRNA Isolation Kit, Epicentre). Then, each sample was amplified and transcribed into fluorescent cRNA along the entire length of the transcripts without 3′ bias utilizing a random priming method (Arraystar Flash RNA Labeling Kit, Arraystar). The hybridized arrays were washed, fixed, and scanned using the Agilent DNA Microarray Scanner (part number G2505C). Meanwhile, the Arraystar Rat circRNA Array V1 analysis of the liver samples was conducted. Total RNAs were digested with Rnase R (Epicentre, Inc.) to remove linear RNAs and enrich circular RNAs. Then, the enriched circular RNAs were amplified and transcribed into fluorescent cRNA utilizing a random priming method (Arraystar Super RNA Labeling Kit; Arraystar). The labeled cRNAs were hybridized onto the Arraystar Rat circRNA Array V1 (8 ×∉15 K, Arraystar). After having washed the slides, the arrays were scanned by the Agilent Scanner G2505C. Agilent Feature Extraction software (version 11.0.1.1) was used to analyze acquired array images. Statistically significant differences in the expression of lncRNAs, circRNAs, and mRNAs were evaluated in the BDDLT group and the control group based on *p*-values and FDR filtering. A fold change ≥ 2.0, *p* < 0.05, and FDR < 0.05 were considered statistically significant. We deposited the microarray data and additional information to the GEO database with accession number GSE14520 and GSE119748, respectively.

### 2.6. Gene Ontology (GO) and KEGG Pathway Analysis

Gene ontology (GO) analysis provides a controlled vocabulary to describe gene and gene product attributes in any organism. We use topGO to analyze differential genes in order to infer their molecular functions (http://www.geneontology.org). The 10 items with the smallest *p*-values were selected for graphical display; in the case that fewer than 10 items met the statistical criteria, all the items were displayed. We performed KEGG pathway analysis to harvest pathway clusters covering our knowledge on the molecular interaction and reaction networks in differentially regulated gene profiling. Again, the 10 items with the smallest *p*-values were selected for graphical display or all the items if fewer than 10 met the statistical criteria. We also used differentially expressed genes for pathway analysis to infer the pathways they participate in. Pathway analysis is a functional analysis mapping genes to KEGG pathways. The *p*-value (EASE-score, Fisher-*p*-value or Hypergeometric-*p*-value) denotes the significance of the Pathway correlated to the conditions. Lower the *p*-value, more significant is the Pathway. (The recommend *p*-value cut-off is 0.05.) Significant pathways contain some genes that differently, pathway-gene network was constructed by the relationship between pathway and gene.

### 2.7. CNC and CeRNA Network Analysis

CNC is a method of correlation analysis between noncoding RNA and mRNA co-expression data, which is based on the Pearson correlation coefficient (PCC) between coding genes and noncoding transcripts according to their expression levels. The criteria for significant effects were set to PCC ≥ 0.90, *p* < 0.01, and FDR <0.01. The normalized signal intensities of specific mRNA and lncRNA expression levels were used to construct coexpression network. According to the expression data of coding and noncoding RNA, we calculated the co-expression relationship (Pearson correlation coefficient, PCC) between coding and noncoding RNA to construct the network. By analyzing the functions of these known RNA molecules, we can associate unknown RNA molecules with specific signaling pathways or diseases to predict their functions and mechanisms. The network was visualized using Cytoscape software (version 2.8.3) [29]. By analyzing the functions of these known RNA molecules, we can associate unknown RNA molecules with specific signaling pathways or diseases to predict their functions and mechanisms.

As is known to us, the ceRNA regulatory mechanism is very important between the mRNA and ncRNAs, including miRNA, lncRNA, and circRNA. Possible miRNA response elements were searched for based on the lncRNA, circRNA, and mRNA sequences. We predicted target mRNAs and lncRNAs (circRNAs) of these miRNAs based on TargetScan 7.2 and compared these target genes to the differentially expressed genes that were identified in our array results. Then the overlapping of the same miRNA seed sequence binding site both on the lncRNAs/circRNAs, and the mRNA predicted lncRNA/circRNA-miRNA-mRNA interaction.

### 2.8. Cis- and Trans-Regulation Prediction

lncRNAs whose expression showed significant correlations with the cis- and trans-regulation of mRNAs were processed. For cis-regulation, previous study defined that a cis-regulator is the one that exerts its function on a neighbouring gene located at the same chromosome, so we search for the adjacent genes less than 300 kb upstream or downstream of lncRNAs may serve as potential targets for cis-regulation of lncRNA [28]. For trans-regulation, we were more interested in the way lncRNAs exert their effects via transcription factors (TFs). The TFs were predicted by the TRANSFAC8.3 database based on promoter regions of lncRNA. LncRNA binding transcription factors were predicted by scoring and *E* value <0.01. Therefore, we enriched those mRNAs co-expressed with lncRNAs that significantly overlapped with the target genes of a given TF and constructed an lncRNA-TF-mRNA network. The enrichment and connectivity was based on Position Frequency Matrix (PFM) performed as described previously [30, 31].

### 2.9. Statistical Analysis

All statistical analyses were performed in SPSS 21.0 and GraphPad Prism software. Data are expressed as mean ± standard deviation (means ± SD). Statistical significance was defined as *p* value less than 0.05 using two-tailed unpaired Student's *t*-test.

## 3. Results

### 3.1. Brain Death Promotes Early Liver Graft Injury in Rats

**Figure 1:**
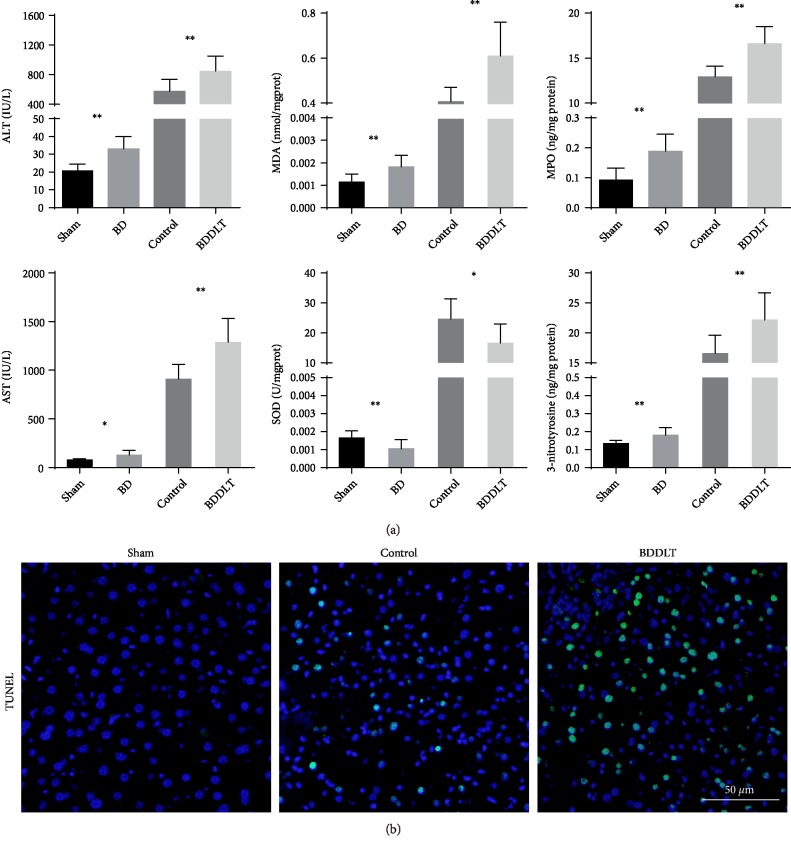
Biochemical and ELISA measurements of liver damage markers; TUNEL staining of liver tissue. (a) ALT, AST, MDA, 3-nitrotyrosine, and MPO levels were significantly increased, and the SOD level was significantly decreased in the BD and BDDLT groups. All data are shown as mean ± SD. The levels of statistical significance are indicated as ^∗^*p* < 0.05, ^∗^^∗^*p* < 0.01 statistically significant. For statistical analysis, a two-tailed Student's *t* test was used. (b) TUNEL staining of liver tissue showed more apoptotic cells in the BDDLT group (scale bar: 50 *µ*m).

Brain death is characterized by a rapid increase in blood pressure (autonomic storm), followed by a decrease, usually to baseline level or below, and a flat-lined EEG (Supplementary [Supplementary-material supplementary-material-1]). We first measured the serum levels of transaminases (AST and ALT). Compared with those in the control group, AST and ALT levels were significantly increased in the BD group and the BDDLT group. Based on the ELISA results, the BD and BDDLT groups showed greater aggravated oxidative stress and inflammatory injury than the control group, characterized by significantly increased levels of MDA, MPO, and 3-notrotyrosine and decreased SOD levels in the BDDLT group (Figure 1(a)). In addition, liver tissue cell apoptosis was detected by TUNEL staining; compared with the control group, the BDDLT group had increased cell apoptosis (Figure 1(b).

### 3.2. Microarray Analysis Reveals Differentially Expressed lncRNA and circRNA Profiles

**Figure 2:**
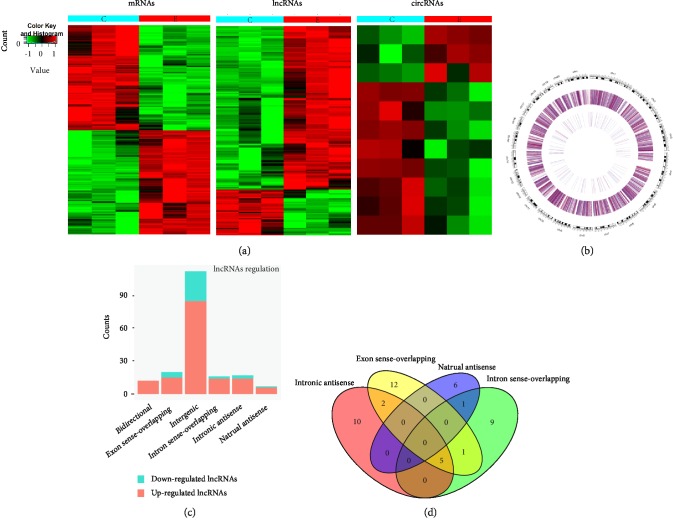
Heat maps showing expression profiles of lncRNAs, circRNAs, mRNAs, and identification of differentially expressed lncRNAs in the liver tissues of the control and BDDLT group. (a) Heat maps show normalized expression values of significantly changed lncRNAs, circRNAs, and mRNAs with a fold change ≥ 2.0, *p* < 0.05, and FDR < 0.05. C: control group tissues. B: BDDLT group tissues. (b) Circos images show the distribution of lncRNA in rat chromosomes, the outermost layer of Circos is the distribution of rat chromosomes, the black and white bands are the cytobands of chromosomes, the middle two circles are the up-down distribution of lncRNA, the intermediate outer ring represents the difference of all detected lncRNAs in the chip, and the intermediate inner ring represents the difference of different lncRNAs (fold change is greater than 2.0, *p* < 0.05). (c) Types and counts of differentially regulated lncRNAs detected by microarray (fold change ≥ 2.0, *p* < 0.05, and FDR < 0.05). The lncRNAs are classified into 6 types according to the relationship and genomic loci with their associated coding genes. (d) The Venn diagram represents the overlapping of relationships and the numbers indicate the lncRNA counts.

Microarray probes successfully detected thousands of transcripts in the liver tissue in the BDDLT group and in the control group. Compared with those in the control group, 228 differentially expressed lncRNAs and 330 differentially expressed mRNAs were detected in three liver tissues in the BDDLT group. Among these differentially expressed transcripts, there were 181 up-regulated lncRNAs, among which LOC102553824 showed the largest difference (fold change: 7.86); there were 47 down-regulated lncRNAs, among which AABR06018038.2 showed the largest difference (fold change: 4.32). There were 164 and 166 up- and down-regulated mRNA transcripts, respectively (fold change ≥ 2.0, *p* < 0.05, and FDR < 0.05). In addition, a total of 5,501 up-regulated and 5,329 down-regulated circRNAs were detected in the liver tissue chips from both the BDDLT and control groups. Coding gene expression profiling revealed 19 mRNAs with a more than 5-fold difference, among which 12 were up-regulated, with the largest fold change being that for Acpp (fold change: 12.22), and 7 were down-regulated, with the largest fold change being that for Serpina7 (fold change: 7.71). Clustering analysis showed that the expression patterns of lncRNAs, mRNAs, and circRNAs significantly differed between the two groups. Thus, expression levels of the identified lncRNAs, cirRNAs, and mRNAs in the liver tissue after BDDLT were significantly different from those in the control group (Figure 2(a)).

These lncRNAs are widely distributed on all chromosomes including the X chromosome and the Y chromosome. Circos images show the distribution of lncRNA in rat chromosomes. The outermost layer of Circos shows the distribution of rat chromosomes. The black and white bands represent the cytobands of the chromosomes. The two circles in the middle represent the up-down distribution of lncRNAs. The more intense the red color, the higher the up-down multiple, and the more intense the blue color, the higher the down-down multiple. The intermediate outer ring represents the difference in all detected lncRNAs in the chip, and the intermediate inner ring represents the difference in different lncRNAs (fold change is greater than 2.0, *p* < 0.05) (Figure 2(b)). The abnormally expressed lncRNAs were divided into six categories according to their relationships with protein-encoding genes: exon-overlapping (10.9%), intron-overlapping (8.7%), natural antisense (3.8%), and intron antisense (9.3%), and there were a number of crossover events among these four categories, with 60.7% being intergenic and 6.6% being bidirectional. Furthermore, intergenic lncRNAs were the largest category among all of the differentially expressed lncRNAs (60.7%), with both the up-regulated intergenic lncRNAs (45.9%) and down-regulated intergenic lncRNAs (14.8%) accounting for large proportions (Figure 2(c)). The Venn diagram presents the overlapping of relationships and the numbers indicate the lncRNA counts (Figure 2(d)).

### 3.3. GO and KEGG Pathway Analysis

**Figure 3:**
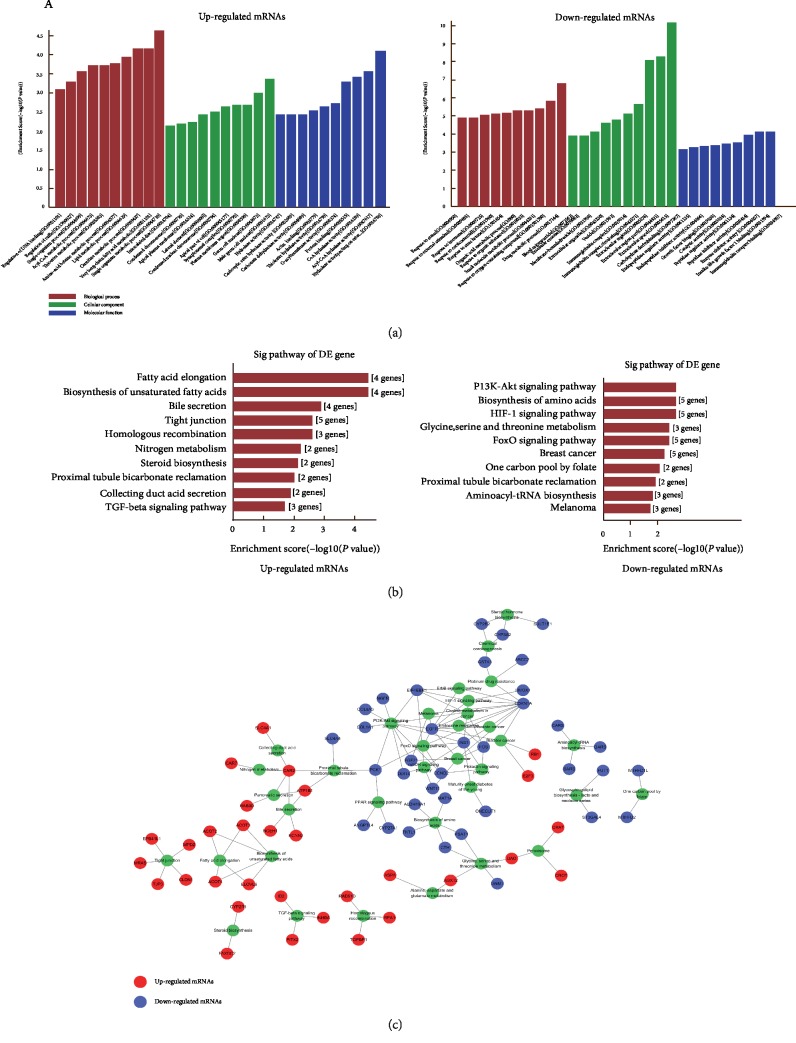
Gene Ontology (GO) and KEGG pathway analysis with top 10 enrichment score. (a) GO analysis of up- and down-regulated mRNAs with top ten enrichment score covering domains of biological processes, cellular components, and molecular functions. (b) KEGG pathway enrichment analysis of up- and down-regulated mRNAs with top ten enrichment score. (c) The link and overlapping of associated molecules among significant pathways.

GO enrichment analysis of significantly differentially expressed mRNAs can reveal the role of differentially regulated lncRNAs. Up-regulated mRNAs have been found to be primarily involved in single-organism metabolic processes and activities of the germ cell nucleus and hydrolase. Furthermore, down-regulated transcripts mainly participate in drug metabolism, extracellular space, and immunoglobulin receptor binding (Figure 3(a)). Our data revealed ten pathways associated with the up- and down-regulation of mRNAs. KEGG enrichment analysis of the up-regulated protein-coding genes showed that the signaling pathway responsible for fatty acid elongation was the most significantly enriched. In contrast, KEGG analysis of the down-regulated transcripts revealed that the PI3K-Akt signaling pathway was the most significantly enriched pathway (Figure 3(b)). According to the relationship between enrichment pathway and mRNA, the corresponding pathway-gene network map was constructed (Figure 3(c)).

### 3.4. Verification of Differentially Expressed lncRNAs and mRNAs

**Figure 4:**
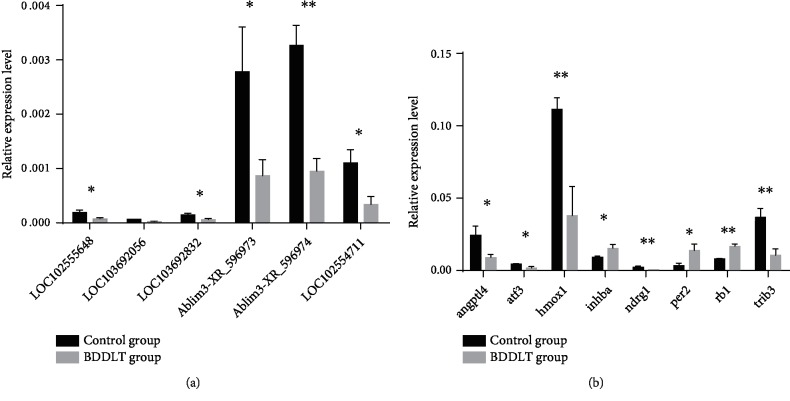
Validation for the expression of significant transcripts by qRT-PCR. The relative expression levels of six lncRNAs (a) and eight mRNAs (b) are shown in the control and BDDLT group liver tissues. All data are shown as the mean ± SD. The levels of statistical significance are indicated as ^∗^*p* < 0.05, ^∗∗^*p* < 0.01. For statistical analysis, a two-tailed Student's *t* test was used.

In our results, mRNAs reported to be involved in hepatic ischemia-reperfusion injury, and their corresponding LncRNA by bioinformatics speculation were selected for qRT-PCR analysis to verify the microarray results, using 10 pairs of liver tissues obtained after LT in the BDDLT group and the control group. The analysis revealed that expression of LOC102555648, LOC103692056, LOC103692832, Ablim3-XR_596973, Ablim3-XR_596974, and LOC102554711 decreased in the liver tissue of the BDDLT group, consistent with the microarray results (Figure 4(a)). Similarly, consistent with the microarray results, eight mRNAs showed altered expression compared with those in the control group, with Per2, Inhba, and Rb1 up-regulated, but Angptl4, Atf3, Hmox1, Ndrg1, and Trib3 down-regulated (Figure 4(b)).

### 3.5. CNC and Functional Prediction of lncRNAs/mRNAs

**Figure 5:**
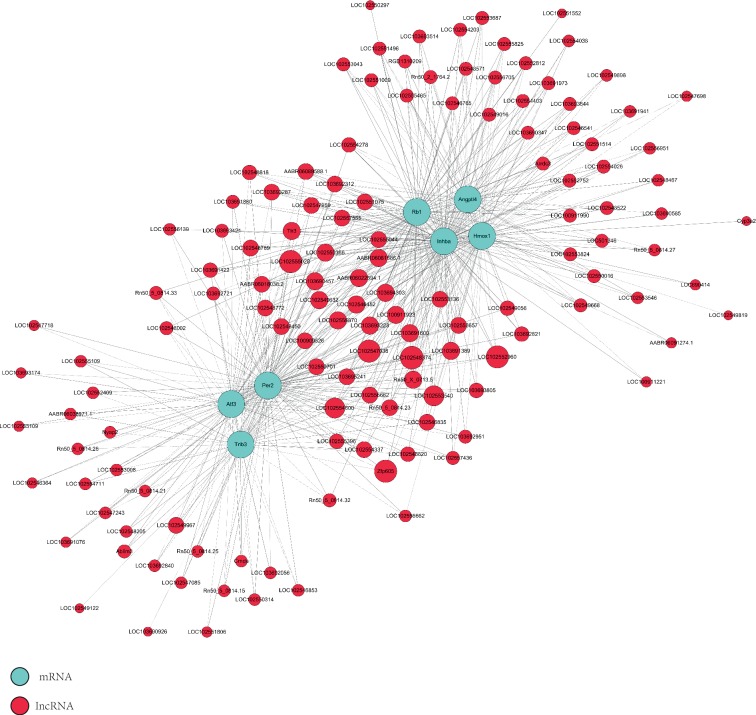
Coding noncoding gene co-expression networks of 7 significant mRNAs with their associated lncRNAs. The network is based on the Pearson correlation coefficient (the absolute value of PCC ≥ 0.90, *p*-value <0.01, and FDR < 0.01). Solid lines indicate positive correlations, and dashed lines indicate negative correlations. The size of the lncRNA dot represents the number of genes co-expressed with the lncRNA.

We selected seven coding genes with significant differential expression to construct the CNC network based on the correlation degree. These mRNAs are involved in many biological processes such as the cell cycle, apoptosis, epithelial-mesenchymal transition (EMT), and angiogenesis. The network revealed that the up-regulated lncRNA AABR06081886.1 was negatively correlated with Atf3, Hmox1, Angptl4, and Trib3, whereas the down-regulated lncRNA LOC100911923 was positively correlated with these four protein-coding genes, which are mainly involved in cell apoptosis (Figure 5).

### 3.6. Prediction of Cis- and Trans-Regulatory Functions of lncRNAs (Adjacent Coding Genes of lncRNAs and lncRNA-TF-mRNA)

**Figure 6:**
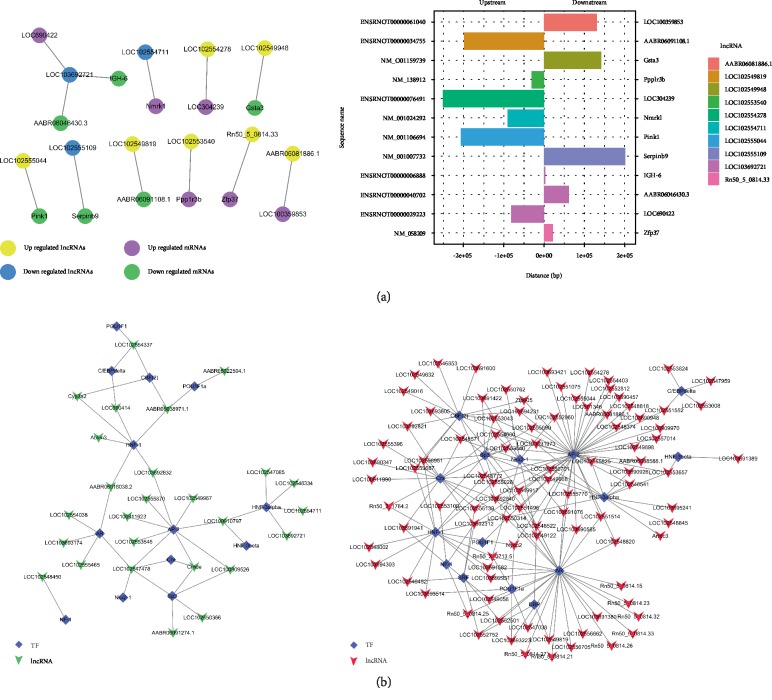
Cis regulation of lncRNAs to nearby coding genes and the network of enrichment transcription factors and query lncRNAs. (a) lncRNAs and their potential cis regulated nearby genes are shown in the network and the distances between lncRNAs and their cis regulated genes are presented. (b) According to the prediction of transcription factor regulation in the promoter region of lncRNA, the transcription factors of rats in the TRANSFAC8.3 database were predicted. The up-regulated lncRNAs-TFs network consists of 15 TFs and 95 correlated lncRNAs, and they are connected by 172 edges. The down-regulated lncRNAs-TFs network consists of 13 TFs and 26 correlated lncRNAs, and they are connected by 43 edges.

Based on the coexpression, we further explored how the abnormally regulated lncRNAs could achieve cis- or trans-regulation of mRNAs. Ten differentially expressed lncRNAs were selected to conduct searches for their adjacent coding genes. The coexpressed protein-coding genes were defined as cis-regulated genes with one differentially expressed lncRNA within 300 kb on the same chromosome. The number of adjacent coding genes differs among lncRNAs. For example, LOC103692721 has up to three adjacent coding genes, whereas other lncRNAs have only one target gene. The adjacent gene pattern showed that the down-regulated IGH-6 and ABR06046430.3 were cis-regulated by lncRNA LOC103692721 and involved in liver injury after LT from BD donors. The up-regulated lncRNAs-TFs network consisted of 15 TFs and 95 correlated lncRNAs, and they were connected by 172 edges. The down-regulated lncRNAs-TFs network consisted of 13 TFs and 26 correlated lncRNAs, and they were connected by 43 edges (Figures 6(a) and 6(b)).

**Figure 7:**
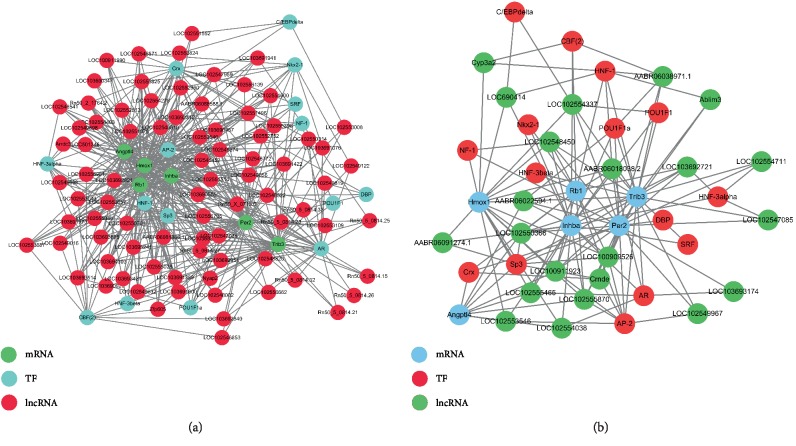
The lncRNA-TF-gene trans regulation network. Network diagram based on the binary analysis of TF-lncRNA using the co-expressed mRNA analysis of lncRNA and mRNA regulated by these transcription factors, using the threshold of abs (PCC) ≥ 0.9 and FDR ≤ 0.05. (a) The up-regulated lncRNAs-TFs-genes network consists of 83 lncRNAs, 15 TFs, and 6 correlated genes. (b) The down-regulated lncRNAs-TFs-genes network consists of 22 lncRNAs, 15 TFs, and 6 correlated genes.

Assuming that lncRNAs have transregulatory functions, we analyzed the mRNAs coexpressed with these lncRNAs and mRNAs regulated by TFs. We found that the up-regulated-lncRNA-TF-gene network consisted of 83 lncRNAs, 15 TFs, and 6 correlated genes. The down-regulated-lncRNA-TF-gene network consisted of 22 lncRNAs, 15 TFs, and 6 correlated genes. The lncRNA-TF-mRNA diagram showed that the lncRNA LOC102553657 trans-regulates HMOX1 via the HNF-3beta TF. Next, on the basis of TF-lncRNA binary analysis, we additionally introduced mRNAs to construct the TF-lncRNA-mRNA ternary network. Most lncRNAs, including AP-2, AR, HNF-1, Crx, Sp3, CBF (2), HNF-3alpha, Nkx2-1, and POU1F1a, were found to be involved in TF regulation (Figures 7(a) and 7(b)).

### 3.7. Construction of the ceRNA Network

**Figure 8:**
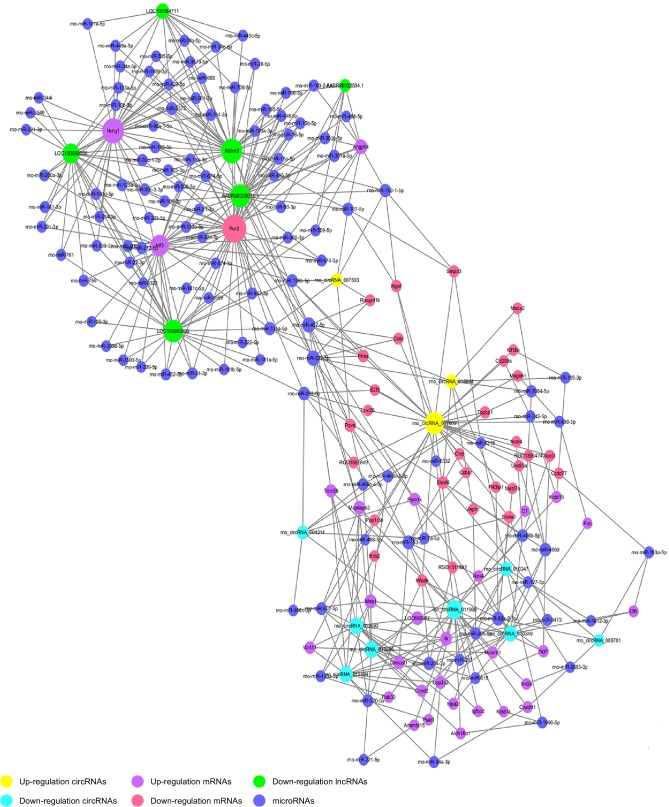
Competing endogenous RNA network. The competing endogenous RNA network is based on lncRNA/miRNA, circRNA/miRNA, and miRNA/mRNA interactions. The edges represent sequence matching, and lncRNAs or circRNAs connect expression correlated with mRNAs via miRNAs.

The ceRNA network was constructed based on microarray results from liver tissues after LT in both the BDDLT group and the control group. Six lncRNAs and 11 circRNAs with significant differential expression were selected. Some of these shared miRNA response elements (MREs); for example, lncRNAs LOC103692832 and rno_circRNA_007609 are ceRNAs of rno-miR-135a-5p targeting Atf3, Per2, and Mras, whereas lncRNAs Ablim3 and rno_circRNA_013693 are ceRNAs of rno-miR-322-5p targeting Per2, Mapkapk3, and Trim59 (Figure 8).

## 4. Discussion

Our study corroborates previous research demonstrating that the complex pathophysiological changes occurring during brain death have a significant impact on graft function and survival [32, 33]. The “autonomous storm” during brain death is considered the main cause of hemodynamic instability, which can lead to insufficient organ perfusion, activate proinflammatory factors, and thus promote liver injury soon after transplantation [34, 35]. We found that the levels of transaminases were elevated after BDDLT, along with activation of inflammatory factors and an increased number of apoptotic cells. However, the mechanisms underlying liver injury after BDDLT remain unclear.

The traditional view of gene regulation focuses on protein-coding genes; however, this view has changed since the discovery of many noncoding genes such as lncRNAs, circRNAs, and microRNAs [9]. The roles that lncRNAs can play in the diagnosis and treatment of liver diseases have been recognized [36]. Abnormal lncRNA expression levels have been found in different types of liver injury, suggesting that lncRNAs are involved in the pathogenesis of liver injury [37, 38]. MEG3 and GAS5 can inhibit liver fibrosis, while TUG1 can protect liver grafts during cold preservation [39–41]. A potential relationship between liver injury and circRNA has been previously established, suggesting that circRNA inhibits hepatic steatosis [42]. A large number of differentially expressed circRNAs were also found in hepatic ischemia-reperfusion injury models [21]. In this study, we analyzed for the first time the changes in expression of lncRNAs, circRNAs, and mRNAs in liver tissues after BDDLT.

We obtained the expression profiles of lncRNAs and circRNAs by microarray analysis. A large number of differentially expressed lncRNAs and circRNAs were found in liver tissues after BDDLT, with about 60% of the down-regulated lncRNAs being intergenic. Six down-regulated lncRNAs and eight differentially expressed mRNAs were further verified by qRT-PCR. Overall, the qRT-PCR verification results were consistent with the microarray results. The differentially expressed genes were subsequently classified according to the data using thermograms and cluster maps. We also found that the differentially expressed lncRNAs were distributed across all the chromosomes, suggesting that every chromosome in the liver tissue, including the X and Y chromosome, was abnormal after BDDLT. We found that Hmox1, Ndrg1, and Atf3 were significantly down-regulated in liver tissues after BDDLT; thus, these genes may be involved in inducing apoptosis after BDDLT to cause liver injury.

KEGG pathway analysis of differentially expressed mRNAs revealed that multiple pathways (primarily the fatty acid elongation pathway and the PI3K-Akt signaling pathway) were involved in liver injury after BDDLT. GO analysis revealed that the main mechanisms of liver injury after BDDLT included single-organism metabolism, drug metabolism, and immunoglobulin receptor binding. At present, the functions of most lncRNAs are unknown, and constructing a CNC network is one method for predicting lncRNA functions [43, 44]. We found that the expression levels of many lncRNAs were significantly associated with protein-encoding genes through correlation of expression levels. Therefore, we established a CNC network to predict the relationship between lncRNAs and mRNAs. The network revealed that the up-regulated lncRNA AABR06081886.1 was negatively correlated with Atf3, Hmox1, Angptl4, and Trib3, whereas the down-regulated lncRNA LOC100911923 was positively correlated with these four protein-coding genes, which are mainly involved in cell apoptosis.

It has been reported that lncRNA can affect the expression of adjacent or distal genes via cis-regulation and trans-regulation [45–47]. We studied the relationships between ten significantly differentially expressed lncRNAs and their adjacent genes, in an attempt to provide a new theoretical mechanism for liver injury after BDDLT. We found that the number of adjacent coding genes differed among lncRNAs; for example, LOC103692721 had up to three adjacent coding genes, whereas other lncRNAs had only one target gene. In addition, the adjacent gene pattern showed that the down-regulated gene IGH-6 and ABR06046430.3 were cis-regulated by lncRNA LOC103692721 and involved in liver injury after BDDLT.

Furthermore, based on our results, we constructed the lncRNA-TF network. Transcriptional regulation by lncRNAs can be predicted from the regulation of TFs, which they express [48–50]. The combination of TFs and cis-acting elements can also regulate the expression levels of target genes in the promoter region [51, 52]. It has also been reported that TFs participate in the occurrence and development of liver injury [53, 54]. Therefore, a comprehensive analysis of TFs and differentially coexpressed genes can improve our understanding of liver injury after BDDLT. A number of lncRNAs may be involved in specific pathways for TF regulation. Therefore, assuming that lncRNAs regulate transcription, we analyzed mRNAs coexpressed with lncRNA and TF-regulated mRNAs. Using *p* < 0.01 and FDR <0.01 as the statistical criteria, we found that each lncRNA was associated with between one to over a dozen TFs, and that each lncRNA-TF pair resulted from the enrichment of several genes, providing key data for subsequent studies. In the liver tissues from the BDDLT group, we found that the up-regulated lncRNAs-TFs network consists of 15 TFs and 95 correlated lncRNAs, and they are connected by 172 edges. The down-regulated lncRNAs-TFs network consists of 13 TFs and 26 correlated lncRNAs, and they are connected by 43 edges. Furthermore, the lncRNA-TF-mRNA diagram showed that lncRNA LOC102553657 trans-regulated HMOX1 via HNF-3beta TF. Thus, we speculate that these TFs participate in liver injury after BDDLT by regulating the transcription of lncRNAs and mRNAs. However, the relationships between lncRNAs and TFs needs further investigation.

Pandolfi et al. introduced a hypothesis called the ceRNA mechanism, which proposed that transcripts such as mRNAs, pseudogenes, and lncRNAs can serve as natural miRNA sponges by competitively binding to miRNA response elements (MREs) to suppress their expression and function [55]. Indeed, ceRNAs have important influences on gene expression at the post-transcriptional level, and several studies have reported that lncRNA is involved in the pathogenesis of liver injury through ceRNA [56–59]. However, the roles of lncRNA- and circRNA-related ceRNAs in liver injury have not been studied. In the current study, we constructed for the first time the ceRNA network with lncRNA-miRNA-circRNA-mRNA associations based on microarray results. Our results reveal a specific ceRNA network underlying liver injury after BDDLT. We found that the lncRNAs LOC103692832 and rno_circRNA_007609 were ceRNAs of rno-miR-135a-5p targeting Atf3, Per2, and Mras, whereas the lncRNAs Ablim3 and rno_circRNA_013693 were ceRNAs of rno-miR-322-5p targeting Per2, Mapkapk3, and Trim59. Thus, lncRNAs and circRNAs harbor MREs and thus play key roles in liver injury after BDDLT.

## 5. Conclusions

We found that brain death promotes liver injury after LT. Furthermore, we identified a number of specific abnormally regulated lncRNAs and circRNAs, which could be promising markers of the pathogenesis of liver injury after BDDLT and provide new directions for predicting and diagnosing this condition. Our research paves the way for further studies regarding the roles of lncRNAs and circRNAs after BDDLT.

## Figures and Tables

**Table 1:** Primers designed for qRT-PCR validation of candidate lncRNAs and mRNAs.

Gene	Primer	Annealing temperature (℃)	Product length (bp)
GAPDH (RAT)	F:5′ GCTCTCTGCTCCTCCCTGTTCTA 3′	60	124
	R:5' TGGTAACCAGGCGTCCGATA 3′		
Inhba	F:5′ AACGGGTATGTGGAGATAGA 3′	60	239
	R:5′ TGAAACAGACGGATGGTG 3′		
Per2	F:5′ TTTTTCTGCCGTGTCAGTGTT 3′	60	213
	R:5′GTTTGGTGTGTGGGTTGTTGT 3'		
Rb1	F:5′ AAATCATCGTAACTGCGTAT3′	60	104
	R:5′GTAGAACACTATAATGGAATCAAAC 3′		
Trib3	F:5′ CAAGTTGCGTCGATTTGTC 3′	60	245
	R:5′ CAGAGTCCTGGAACGGGTAT 3′		
Hmox1	F:5′ GGTCCTGAAGAAGATTGCG3′	60	258
	R:5′GAGGGACTCTGGTCTTTGTG 3′		
Ndrg1	F:5′ CTTCGGCAAGGAGGAGATAC 3′	60	196
	R:5' TCCAACCACGAGCAGAGC 3'		
Angptl4	F:5′ AACGCCACCCGCTTACAC 3′	60	142
	′ AGCCTCCATCTGAAGTCATCTC 3′		
Atf3	F:5′ AATTGCTGCTGCCAAGTGT 3′	60	184
	R:5′ CTGAGCCCGGACGATACA 3′		
LOC103692832	F:5′ ATGGTTCGGGAGAGTTACTAGC 3′	60	103
	R:5′ ATGGTTCCAGAGGGATAAGAGT 3′		
LOC102555648	F:5′ CGATCTAAAACTTGTCCGAACA 3′	60	87
	R:5′ CAGCTACTAAACCCGTCCGT 3′		
LOC102554711	F:5′ AATGACGGATGAGCCGATAC 3′	60	66
	R:5′ CGCCCAAGTAGTTGTTGCA 3′		
Ablim3-XR_596974	F:5′ TACCGCTCAGGTGATTTGTCC 3′	60	205
	R:5′ GCATACTGCGGTCAAAATCG 3′		
Ablim3-XR_596973	F:5′ TACTACCGCTCAGCTGGAGAAA 3′	60	99
	R:5′ TTCACTCGTCTTGCTCTTGGTT 3′		
LOC103692056	F:5′ TGGACACTGGGGAGACATT 3′	60	175
	R:5′ AAACTGCTCCATGATTGCTG 3′		

## Data Availability

The data used to support the findings of this study are available from the corresponding author upon request.

## Supplementary Materials

Supplementary Materials Supplementary Figure 1: EEG (A) and MAP (B) 6 hours after brain death.
