# Supplementary figures and images for: Microarray Analysis For Expression Profiles of lncRNAs and circRNAs in Rat Liver after Brain-Dead Donor Liver Transplantation
Source: Biomed Res Int. 2019 Nov 7;2019:5604843. doi: 10.1155/2019/5604843 (PMC6881575; doi:10.1155/2019/5604843)

Supplementary Materials

Supplementary Figure 1: EEG (A) and MAP (B) 6 hours after brain death.

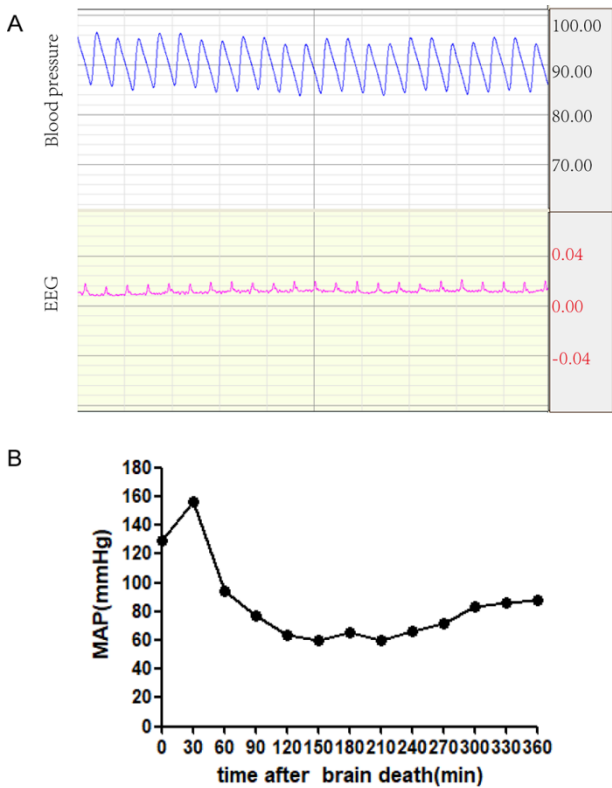

Supplement: Supplementary Materials — Supplementary Figure 1: EEG (A) and MAP (B) 6 hours after brain death. [file 5604843.f1.pdf]
